# Algorithms for predicting COVID outcome using ready-to-use laboratorial and clinical data

**DOI:** 10.3389/fpubh.2024.1347334

**Published:** 2024-05-14

**Authors:** Alice Aparecida Lourenço, Paulo Henrique Ribeiro Amaral, Adriana Alves Oliveira Paim, Geovane Marques-Ferreira, Leticia Gomes-de-Pontes, Camila Pacheco Silveira Martins da Mata, Flávio Guimarães da Fonseca, Juan Carlos González Pérez, Jordana Grazziela Alves Coelho-dos-Reis

**Affiliations:** ^1^Laboratório de Virologia Básica e Aplicada, Instituto de Ciências Biológicas, Departamento de Microbiologia, Universidade Federal de Minas Gerais, Belo Horizonte, Brazil; ^2^Departamento de Física, Instituto de Ciências Exatas, Universidade Federal de Minas Gerais, Belo Horizonte, Brazil; ^3^Hospital Risoleta Tolentino Neves, Universidade Federal de Minas Gerais, Belo Horizonte, Brazil; ^4^CT Vacinas, Universidade Federal de Minas Gerais, Belo Horizonte, Brazil

**Keywords:** SARS-CoV-2, COVID-19, hematological and biochemical parameters, predictive biomarkers, machine learning

## Abstract

The pandemic caused by severe acute respiratory syndrome coronavirus 2 (SARS-CoV-2) is an emerging crisis affecting the public health system. The clinical features of COVID-19 can range from an asymptomatic state to acute respiratory syndrome and multiple organ dysfunction. Although some hematological and biochemical parameters are altered during moderate and severe COVID-19, there is still a lack of tools to combine these parameters to predict the clinical outcome of a patient with COVID-19. Thus, this study aimed at employing hematological and biochemical parameters of patients diagnosed with COVID-19 in order to build machine learning algorithms for predicting COVID mortality or survival. Patients included in the study had a diagnosis of SARS-CoV-2 infection confirmed by RT-PCR and biochemical and hematological measurements were performed in three different time points upon hospital admission. Among the parameters evaluated, the ones that stand out the most are the important features of the T1 time point (urea, lymphocytes, glucose, basophils and age), which could be possible biomarkers for the severity of COVID-19 patients. This study shows that urea is the parameter that best classifies patient severity and rises over time, making it a crucial analyte to be used in machine learning algorithms to predict patient outcome. In this study optimal and medically interpretable machine learning algorithms for outcome prediction are presented for each time point. It was found that urea is the most paramount variable for outcome prediction over all three time points. However, the order of importance of other variables changes for each time point, demonstrating the importance of a dynamic approach for an effective patient’s outcome prediction. All in all, the use of machine learning algorithms can be a defining tool for laboratory monitoring and clinical outcome prediction, which may bring benefits to public health in future pandemics with newly emerging and reemerging SARS-CoV-2 variants of concern.

## 1 Introduction

The global panorama was abruptly reshaped at the end of 2019, when a new coronavirus, SARS-CoV-2, emerged, heralding the beginning of the COVID-19 pandemic. The virus quickly crossed borders and redefined the way biosciences worked. As nations faced the unprecedented challenges posed by this highly contagious and often serious respiratory disease, a collective call to action to control the infection through the production of an effective vaccine reverberated around the world with the greater aim of containing the virus. From frontline healthcare workers to research laboratories, the pandemic has demanded a comprehensive response.

Several countries started vaccination programs against SARS-CoV-2, totalizing over 13 billion doses of vaccines administered by 2023. Nonetheless, despite many efforts to improve vaccine coverage, less than 70% of the world population received at least one dose of these vaccines (1). Moreover, such rates are heterogeneous and may be under 30% in low-income regions. Overall, nearly 5.7 million new cases of COVID-19 were reported at the beginning of 2023 (2).

While most COVID-19 cases may remain asymptomatic or with mild symptoms, patients with severe COVID-19 may present cardiovascular problems, liver, neurological, gastrointestinal, kidney and hematological outcomes (3). In addition, the rate of mortality of critically ill COVID-19 patients without vaccination is high and post-acute sequelae are common in patients who survive the disease. Therefore, it is essential to study and understand the mechanisms involved in mortality and survival of severe COVID-19 as well as developing tools based on ready-to-use laboratorial and clinical data.

Currently, there is no definite tool to predict mortality by COVID-19, although several biomarkers have been proposed for such purpose (4). Tests such as blood count, creatine kinase (CK), D-dimer, lactate dehydrogenase (LDH), C-reactive protein (CRP), aspartate aminotransferase (AST), ferritin, prothrombin, glycemia, ferritin, cardiac biomarkers (troponin, CK-MB, Pro-BNP), 25 OH-Vitamin D, ions (Na/ K/Ca/Mg) and others should be taken into consideration in the diagnosis (5). However, there is no standard protocol, thresholds defined nor algorithms using those parameters to predict clinical outcome.

In this sense, the field of artificial intelligence (AI) and, more precisely, machine learning (ML) has been making remarkable strides in several sectors, demonstrating its potential to revolutionize various aspects of modern medicine. This convergence between the COVID-19 pandemic and the power of AI and ML underlines the importance of interdisciplinary collaboration in tackling complex challenges, offering diverse possibilities in a future when technology and healthcare interconnect to create more resilient, adaptable and efficient global systems.

The World Health Organization has recently called attention to the importance of AI as an aid to the healthcare system and has issued regulatory considerations on artificial intelligence for health (6), which highlights the importance of systems being efficient and safe, as well as being made quickly available to persons in need. In fact, the speed with which this technology is deployed and the possibility of errors during this process must be considered in order to prevent causing any high-scale harm to healthcare professionals and, consequently, patients. Therefore, the regulation of artificial intelligence in health is essential and could bring safe benefits to the population, as an important tool for health promotion and care.

The use of AI and ML in the context of the pandemic is centered on pattern detections that can be obtained from medical images to laboratory parameters. However, AI is not limited to this, since it can be equally used in therapy, prognosis and also extremely useful in public health management (7–9). These tools are invaluable for understanding, predicting, and responding to the spread of COVID-19, demonstrating their ability to provide data-based information and facilitate evidence-based decision-making. In fact, our detection approach meets most of the World Health Organization (WHO) guidelines for point-of-care bioanalysis, including sensitivity, accessibility, ease of use, speed of delivery and rapidity (10, 11). This advantage contributes significantly to the creation of new diagnostic concepts. It should be noted that this strategy is promising for large-scale individual testing, which is essential for an effective response to the pandemic and the gradual restoration of social circulation (12).

This study, therefore, uses machine learning (ML) to predict the clinical outcomes of severe COVID-19 patients, taking advantage of readily available laboratory parameters and clinical data. To achieve this goal, we adopted a new methodological approach, using data from patients in the intensive care unit (ICU) of a central hospital of the metropolitan area of Belo Horizonte, (Minas Gerais state), one of the largest cities in Brazil, which was one of the most affected countries by COVID-19 worldwide. Using conventional and unconventional statistical analysis, survival versus deceased groups were compared by constructing Receiver Operating-Characteristics (ROC) curves to assess the performance and accuracy of each parameter evaluated. Finally, we used cutting-edge strategies based on the Python programming language to develop a prediction solution based on machine learning, a pioneering approach never before applied to this data set. With this, we underline the importance of routine hospital laboratory tests and their integration with appropriate machine learning models, offering another avenue for the early identification of patients in need of immediate intervention.

In the present study, we presented ML-based methods to define and predict the clinical outcome of patients and the importance of using it to classify the severity of COVID-19 patients. Optimal and medically interpretable machine learning algorithms for outcome prediction are presented for each time point. It was found that urea is the most paramount variable for outcome prediction over all three time points. However, the order of importance of other variables changes for each time point, demonstrating the importance of a dynamic approach for an effective patient’s outcome prediction.

The article is organized as follows: in section 2, we present the laboratory parameters we evaluated in the three different times and their correlation shown as heatmaps. We also define which methods we used to analyze the data. In section 3 we present the results obtained from the different methods for analyzing the parameters. In section 4 we discuss the best method for predicting the clinical outcome of patients and the importance of using it to classify the severity of COVID-19 patients. Section 5 shows the limitations of our work.

## 2 Methods

### 2.1 Patient data

This study was carried out using data from patients admitted to the ICU of the Risoleta Neves hospital in Belo Horizonte, Minas Gerais state, Brazil, which is a referral unit for clinical and surgical emergencies managed by the Federal University of Minas Gerais. The study was approved by the Institutional’s Ethics Committee (CAAE: 45086721.1.0000.5149 - opinion number 4.751.423).

The patients were admitted by the hospital between May 2020 and March 2021 and their inclusion in the study was dependent on the confirmation of SARS-CoV-2 infection by RT-PCR. Patients were over 18 years old (median age range = 64) and had hematological and biochemical data accessed at three time points: time 1 (T1–0 to 6 days of hospitalization), time 2 (T2–7 to 14 days of hospitalization) and time 3 (T3 – >14 days of hospitalization).

The COVID patients (total *n* = 81) were further classified according to the outcome of the disease and referred to as: “Discharge” (*n* = 28) or “Death” (*n* = 53). Serum samples were collected in tubes containing gel and in the absence of anticoagulant by venipuncture during the morning routine of the ICU visit, aliquoted and stored at -80°C until processing.

### 2.2 Statistical analysis

GraphPadPrism 8.0 software (GraphPad Software Inc.) was used for the conventional statistical analysis of the data to compare the groups. The Analysis of Variance (ANOVA) test, followed by Tukey’s post-test for parametric data and the Kruskal-Wallis test, followed by Dunn’s post-test for non-parametric data were used to compare the groups. For the comparative analysis between two groups, the Student’s t-test was used for parametric data and the Mann–Whitney test for non-parametric data.

The groups of COVID-19 patients were compared and contrasted in the three time periods evaluated in this study. For that, the Receiver Operating-Characteristics curves or ROC curves were constructed to assess the performance and accuracy of each parameter evaluated, with values of the Area Under the Receiver Operating-Characteristics Curve (AUROC) less than 0.70 showing poor performance, values between 0.70 and 0.80 showing moderate performance, values between 0.80 and 0.90 showing good performance, and values greater than or equal to 0.90 showing excellent performance. For the analyses, the patient’s results were evaluated according to clinical and laboratory factors. In all cases, statistically significant differences were considered when the *p*-value was less than 0.05.

We also used GraphPadPrism 8.0 (GraphPad Software Inc.) to build the correlation amongst all parameters, which were visualized by heatmaps that were built to underscore putative and prospective clusters of parameters with predictive potential. Spearman r correlation indices were the basis to create the heatmaps. The data under scrutiny in the heatmaps were age, outcome (discharge or death), hospital stay, red blood cells, hemoglobin, hematocrit, mean corpuscular volume, global leukocyte count, neutrophils, neutrophil/lymphocyte ratio, eosinophils, basophils, monocytes, lymphocytes, platelets, pH, pCO2, pO2, HCO3, SatO2, BE, potassium, sodium, calcium, chlorine, glycemia, lactate, creatinine, urea, and gender.

### 2.3 Machine learning analysis

In this work, we use the Python language to build a machine learning-based prediction solution. Five different ML models were trained to be able to predict patient’s outcome (discharge or death) with the same data used for statistical analyses for the three time points. These models were Decision Tree Classifier (DT) (13), eXtreme Gradient Boosting (XGBoost) (14), K-Nearest Neighbors (KNN) (15), Logistic Regression (LR) (16), and Support Vector Machine (SVM) (17). With the training of machine learning models, we seek to obtain better results than those obtained by ANOVA test.

The five machine learning models were trained using one to five features. The selection of features was made based on the results of the χ^2^ test, implemented in the Scikit-learn library (18), which evaluates the relationship between random variables, allowing us to identify and exclude the features that are most likely to be unrelated to the class, making them unimportant for the classification. To carry out the categorical data analysis based on χ^2^ test, the missing data was imputed with the median of each feature and scaled in such a way that each feature is in the range from 0 to 1.

The probabilities of patients progressing to death were obtained in a leave-one-out cross-validation (LOOCV) process in which all available samples in the data set are used, one by one, as test data, while the rest of the samples are used as training data. Therefore, in each LOOCV cycle, we have n-1 samples in the training base and 1 test sample, where n is the total number of samples. There are plenty of available cross-validation (CV) techniques. To choose the optimal CV technique, the bias-variance trade-off should be considered, as well as the signal-to-noise ratio of the data, the computational complexity, and the final user’s preferences (19). However, LOOCV is particularly suited for small data sets with high signal-to-noise ratio over CV set or other CV techniques because it provides a model performance estimate that is less susceptible to bias, it tends not to overestimate the test error rate, and there is no randomness in the training/validation database splits (19, 20). LOOCV is computationally expensive but a very powerful and versatile technique, suitable for any kind of predictive model (20). In each LOOCV cycle, we calculate the probabilities of the “death” outcome for the training base and the test sample. Then, with the training base probabilities, we determine the training AUROC in each cross-validation cycle as well as the probability of the “death” outcome of the test sample. At the end of the LOOCV process, we have n training AUROCs and n test sample probabilities. The average training AUROC is the average of the “n” training AUROCs and the test is obtained with the probabilities of each of the test samples. In each cycle of the LOOCV process, the missing data for each feature present in the training base is imputed with its median, and in addition, we balance it using the Synthetic Minority Oversampling Technique (SMOTE) (21). After balancing the training database, we scale it using the Robust Scaler technique implemented in the Scikit-learn library (18).

For the optimization process of the hyperparameters of the models the Optuna library (22) was used in such a way as to maximize the average training AUROC and the AUROC test. This optimization is known as multi-objective since it considers two objective functions. This was done so that the optimized hyperparameters of the models are such that the training AUROC is always greater than the test AUROC to avoid and monitor underfitting and overfitting models.

## 3 Results

### 3.1 Divergent snapshot of clinical, biochemical and hematological parameters according to disease outcome during severe COVID-19

**Figure 1:**
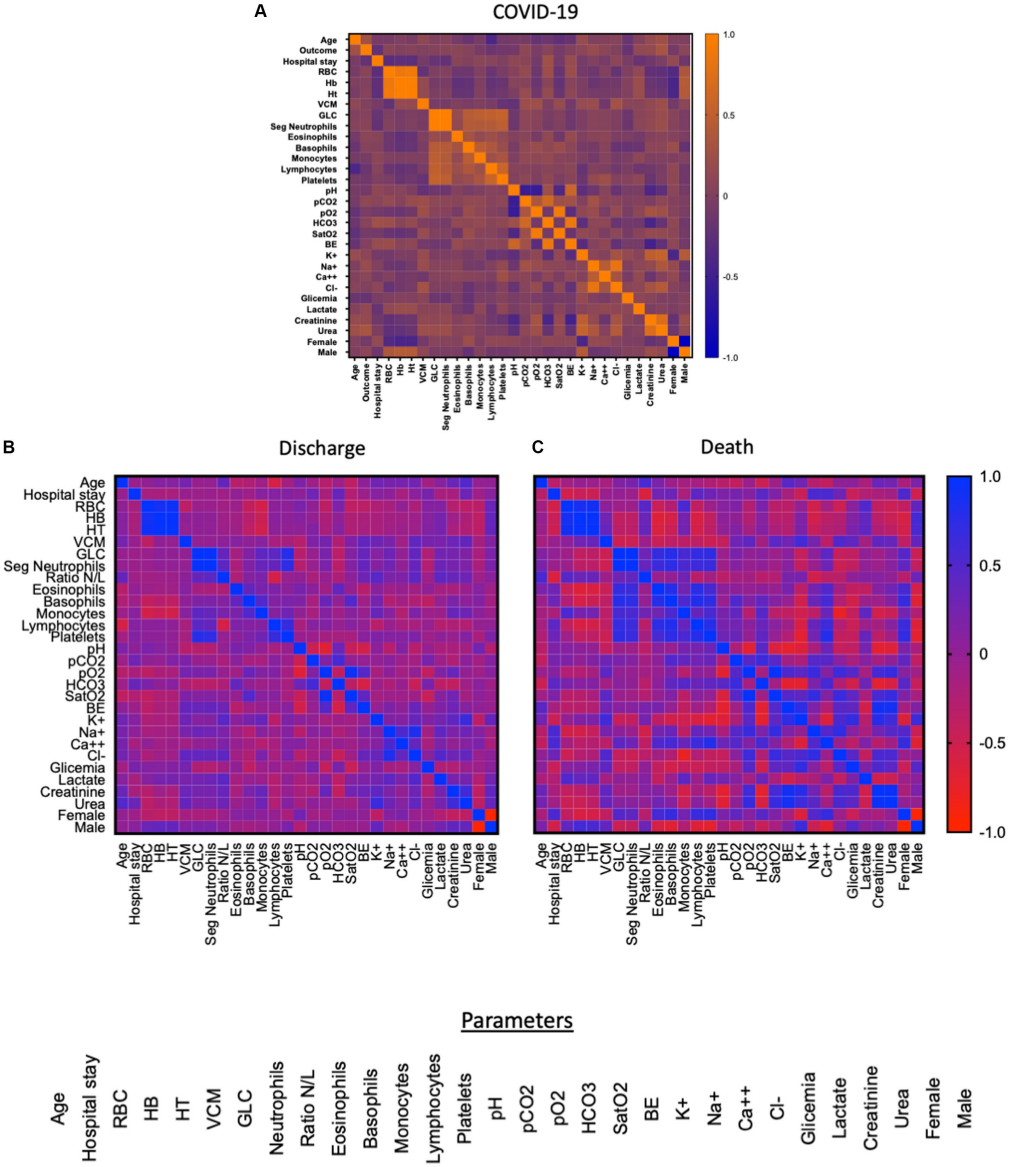
Heatmap of the correlation between the parameters of T1 (0 to 7 days) of the patients’ hospitalization. Red indicates low correlation and blue indicates high correlation. Figure **(A)** shows the correlation between 31 parameters: age, clinical outcome, length of stay, CBR, HB, HT, VCM, GLC, neutrophils, neutrophil/lymphocyte ratio, eosinophils, basophils, monocytes, lymphocytes, platelets, pH, pCO2, pO2, HCO3, SatO2, BE, K+, Na+, Ca++, Cl-, glycemia, lactate, creatinine, urea, gender (female, male) while figure **(B)** refers to the group of patients who were discharged (*n* = 28) and **(C)** refers to the patients who died (*n* = 53), both figures highlight the correlation of 30 parameters: age, length of stay, RBC, HB, HT, VCM, GLC, neutrophils, neutrophil/lymphocyte ratio, eosinophils, basophils, monocytes, lymphocytes, platelets, pH, pCO2, pO2, HCO3, SatO2, BE, K+, Na+, Ca++, Cl-, glycemia, lactate, creatinine, urea, sex (female, male).

In order to provide an overview of the parameters and possibly pinpoint differences between discharge and death outcomes, a comprehensive analysis using heatmap strategy was performed displaying the whole dataset generated by the study. Results are shown in Figure 1 which displays correlations amongst parameters for COVID patients (Heatmap A - Figure 1) as well as the same correlations of patients whose outcomes were either discharge (Heatmap B - Figure 1) and death (Heatmap C - Figure 1). Data analysis at the time of admission (T1) demonstrated that stronger inverse and direct correlations were observed when patients were subdivided by outcome as compared to the COVID-19 group, which displayed less significant correlations in the heatmaps as compared to the subgroups.

**Figure 2:**
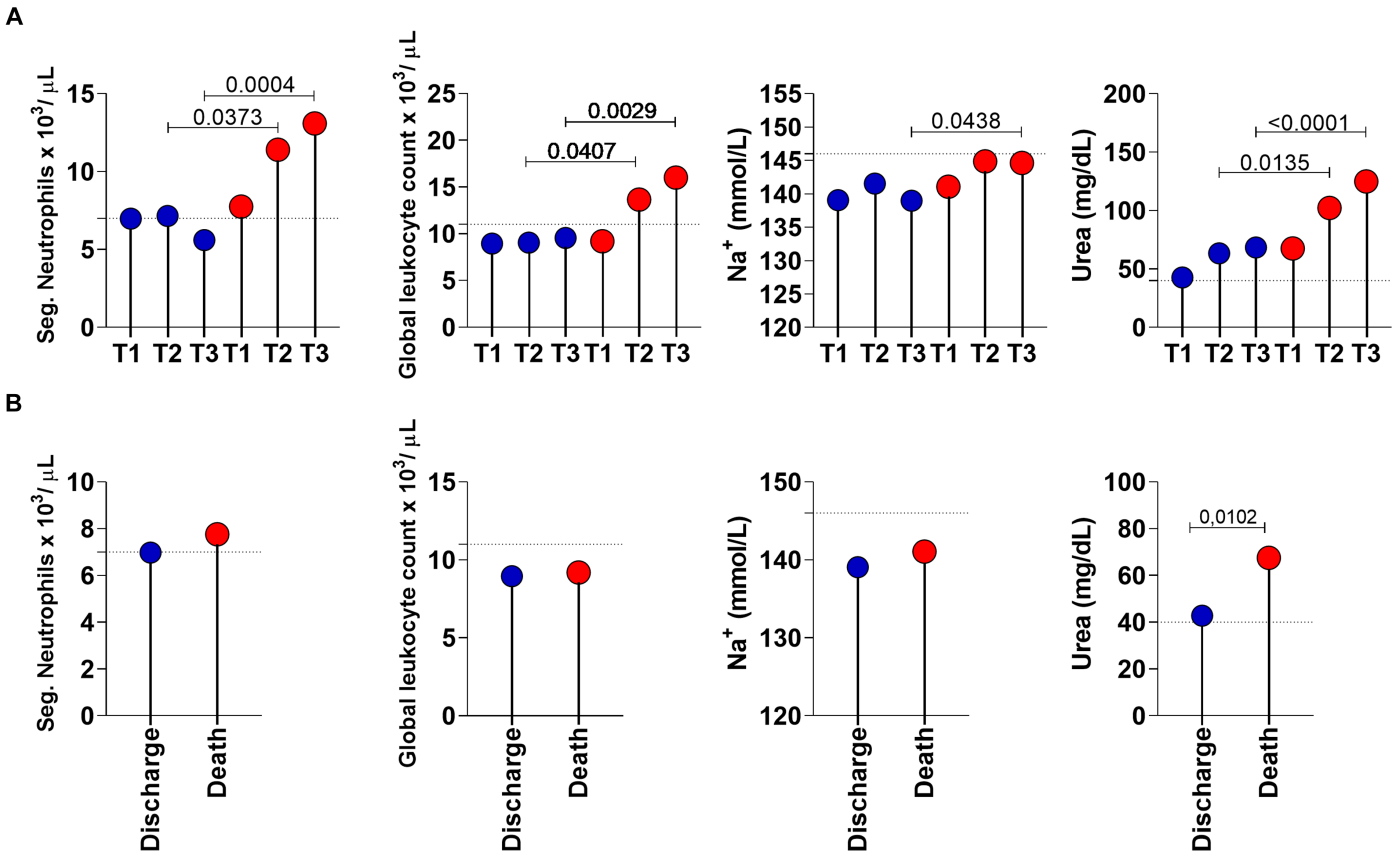
Longitudinal analysis of the selected laboratory parameters in COVID-19 patients with a discharge (*n* = 28; blue circle) and death (*n* = 53; red circle) outcome. **(A)** Lolipop graphs at time point T1 (0 to 6 days of hospitalization), T2 (7 to 13 days of hospitalization) and T3 (greater than 14 days of hospitalization). **(B)** Lolipop graphs at T1. Values of *p* < 0.05 were considered significant and are expressed by * or connector bars. The horizontal traced line represent the Cut-off points: Neutrophils: 7 ×10^3^/ μL; Global leukocytes: 11 ×10^3^ μL; Na^+^: 146 mmol/L and Urea: 40 mg/dL.

Furthermore, the data analysis carried out at the three time points were able to distinguish discharge and death mostly at late time points, starting at T2 for the following parameters: neutrophils, overall leukocytes, sodium and urea. Conversely, urea was the sole parameter able to distinguish patients at an early time point (T1), as shown in Table 1 and Figure 2. The significant *p*-values in Table 1 were obtained by ANOVA followed by Tukey’s post-test when comparing the three times and Student’s *t*-test when comparing two groups (discharge and death).

**Table 1:** Selected laboratory parameters with potential to discriminate disease outcome during severe COVID-19.

Parameters	Time point	Value *p*
Seg. Neutrophils	2	0.0373
Seg. Neutrophils	3	0.0004
Global leukocyte count	2	0.0407
Global leukocyte count	3	0.0029
Na+	3	0.0438
Urea	1	0.0102
Urea	2	0.0135
Urea	3	<0.0001

Figure 2 shows the longitudinal analysis of laboratory parameters considered more important in COVID-19 patients with a discharge (*n* = 28; blue circle) and death (*n* = 53; red circle) outcome. In Figure 2A, the parameters of the two (discharge vs. death) groups are compared at the three time points. In Figure 2B, we observed the same parameters only at T1. Urea (*p*-value = 0.0102) was the only parameter that showed a statistical difference in distinguishing individuals who survived and did not survive COVID-19.

Sodium ion appears below the cut-off point (level considered as normal for healthy individuals) at time points T1, T2 and T3 of discharged patients but only at T1 of patients who progressed to death, with the maximum value for sodium being 146 mmol/L.

Regarding the hemogram analysis, we noticed that the neutrophils of the patients who were discharged are significantly lower than those from patients who progressed to death. The same is observed from T2 time point onwards in the overall leukocyte counts; patients who were discharged had lower overall leukocyte counts than the patients who progressed to death.

### 3.2 Performance of urea to distinguish disease outcome during severe COVID-19

**Figure 3:**
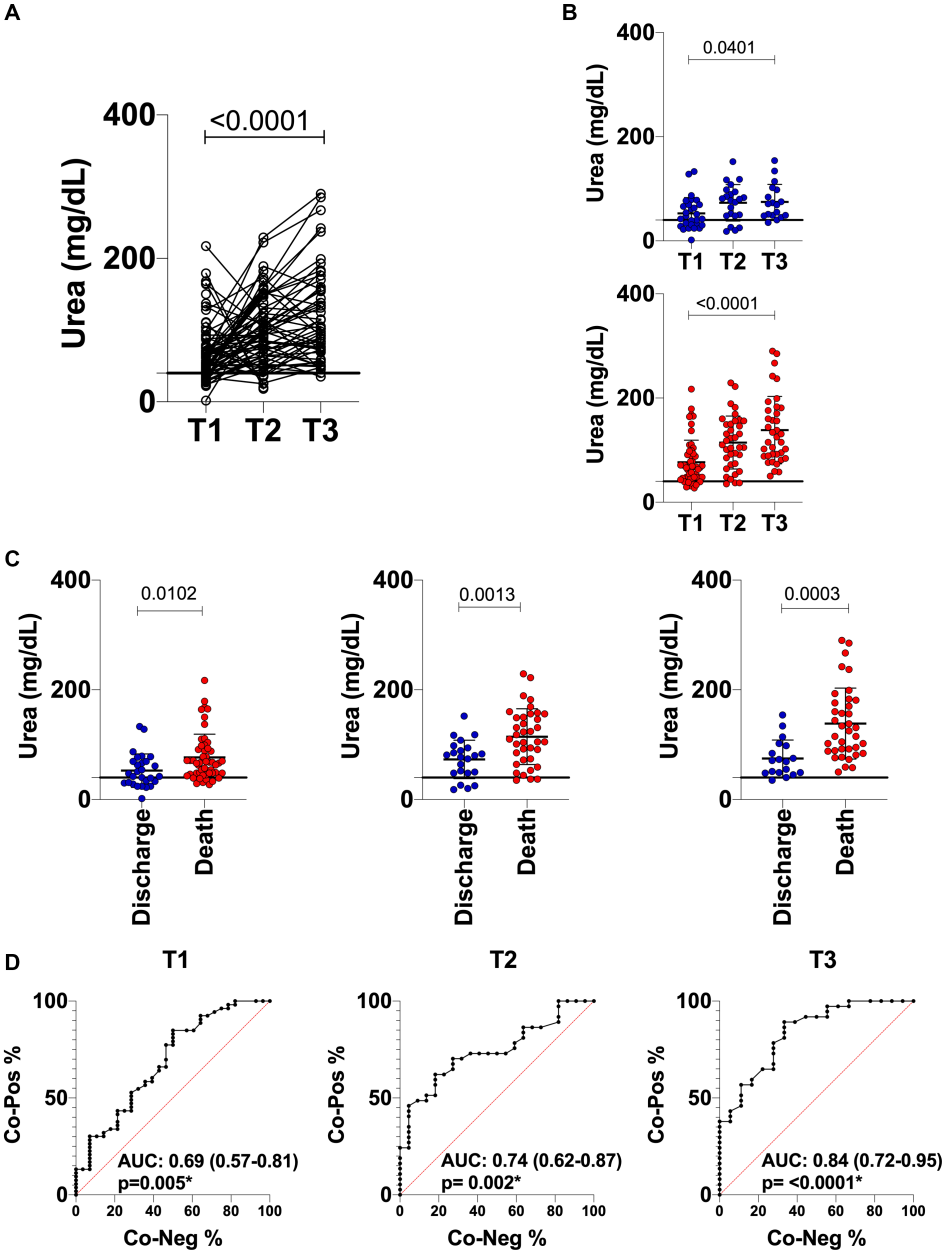
Longitudinal analysis of urea in COVID-19 patients with a discharge (*n* = 26; blue circle) and death (*n* = 43; red circle) outcome. **(A)** Line scatter plots of all COVID-19 patients being followed up (*n* = 69) at time points T1, T2 and T3 (greater than 14 days). **(B)** Scatter plots with individual urea values of COVID-19 patients in collections at time points T1, T2 and T3 with outcome of discharge (*n* = 26; blue circle) and death (*n* = 43; red circle). **(C)** Comparison of discharge versus death for each of the time points evaluated. **(D)** ROC curve analyses showing the performance of urea dosage at each time point of the study. The horizontal traced line represents the Cut-off point of 40 mg/dL for urea. The AUROC in the graph represents the performance of the biochemical parameter in distinguishing discharge and death. Values in brackets in the AUROC correspond to the 95% confidence interval (95%CI).

Considering the interesting results of urea observed, the performance of this parameter in segregating discharge versus death was evaluated using ROC curve analysis. Figure 3 shows the longitudinal analysis for urea using the absolute urea dosage (mg/dL). Individual data analysis demonstrates that urea levels increased over time in patients with COVID-19 regardless of clinical outcome (Figures 3A,B). However, the results confirmed the different pattern between discharge versus death at T1, which is also observed for the late time points (T2 and T3) (Figure 3C). Furthermore, the ROC curves and AUROC values in Figure 3D demonstrate the moderate but always increasing performance of urea as a biomarker of clinical outcome. Therefore, these results indicate that urea should be taken into consideration while building algorithms for prognostics and prediction purposes.

### 3.3 Performance of laboratory parameters to distinguish disease outcome during severe COVID-19 using machine learning approaches

**Figure 4:**
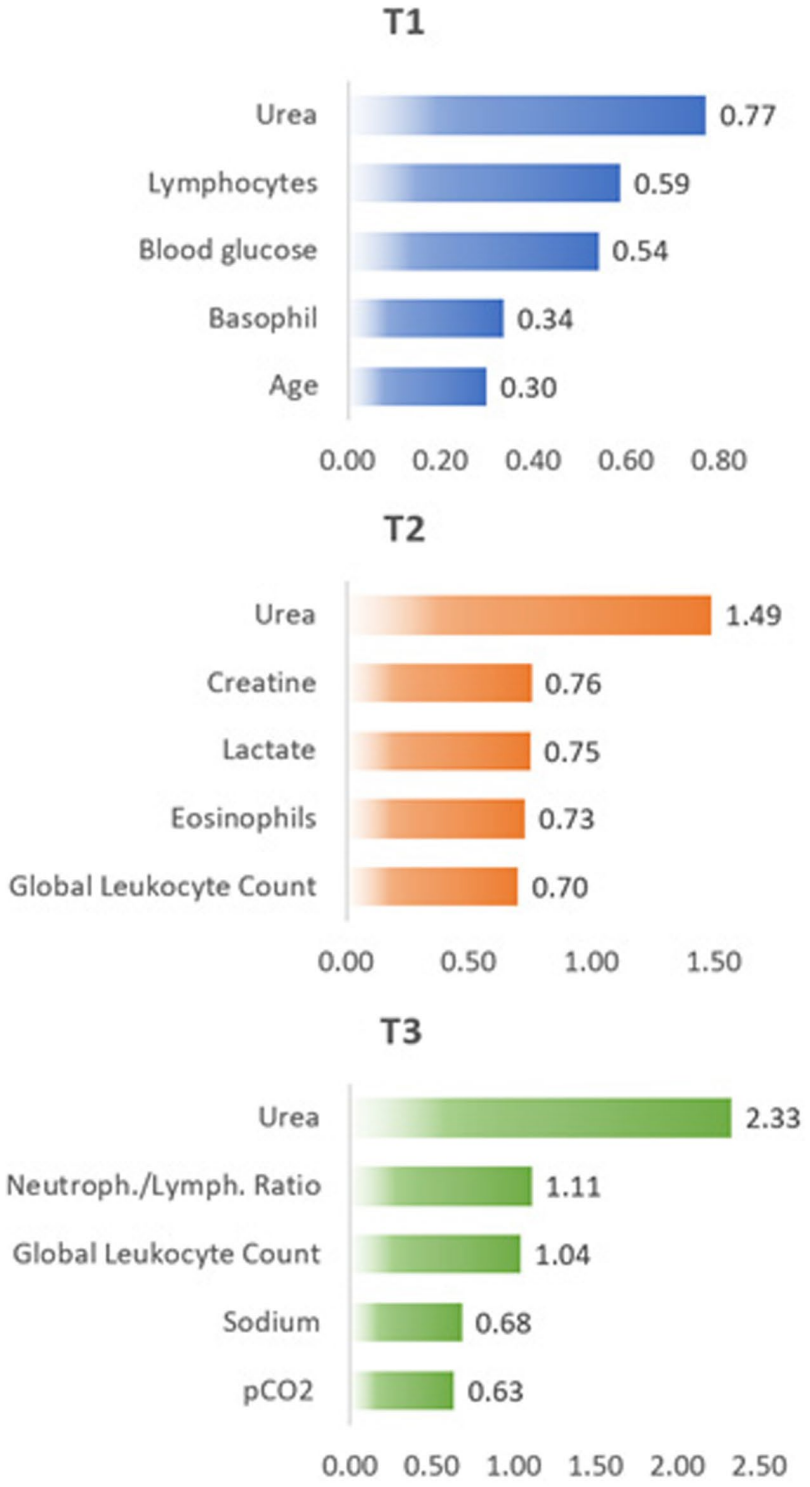
Feature importance results based on χ^2^ test at different time points T1, T2, and T3.

To improve the potency and accuracy of performance analysis of laboratory and clinical parameters of critically ill COVID-19 patients with different clinical outcomes, we performed a feature importance analysis using the same dataset to pinpoint additional biomarkers to discriminate discharge and death. In Figure 4, we show the five more important features for each time point, as selected using the χ^2^ analysis.

In agreement with the above presented results, urea dosage resulted in the most important feature of all parameters considered here, with increasing values as time progressed. However, the remaining four more important features completely changed as time progressed.

Changes in different parameters are directly related to the physiological changes that accompany the development and evolution of the disease and the body’s attempt to recover from the resulting changes. Thus, the increase in creatine in T2 follows the increase in urea concentration already observed in T1 and both are related to the evolution of the patients’ renal failure. The increase in lactate concentration in T2 occurs due to the reduction in oxygen supply in the tissues, showing the advance of cellular dysfunction, which in turn may result from failure of renal functions revealed already in T1 with the increase in urea concentration. The increase in sodium (hypernatremia) is a common effect in the intensive care environment, which justifies the increase in its concentration at T3. Hypernatremia may also be directly associated with the increase in urea concentration (observed in T1 and T2) due to changes in osmotic diuresis that worsen over the period of hospitalization, as well as the increase in lactate concentration (observed in T2).

As described above, we trained and optimized the five different ML models using an increasing number of the most important features, starting from urea, for T1, T2, and T3. Table 2 contains the AUROCs obtained by this procedure. In this table, the highlighted values in gray are the highest results using only the first most important feature (Urea). These results obtained from models trained only with Urea are fundamental because they make a direct comparison with the results obtained by the longitudinal statistical analysis presented previously. The performance of the best ML model considering just urea was moderate for T1 and T2 but reaching a good performance in T3. However, the obtained AUROC values by the best ML model were always higher than by the longitudinal statistical analysis. It is also important to note that the best ML model varies from T1 to T3. While DT resulted in the model with higher performance for T1 [AUROC = 0.78 (0.65–0.91)], it was replaced by SVC in T2 [AUROC = 0.77 (0.64–0.89)], and T3 [AUROC = 0.871 (0.77–0.97)]. The performance of the best ML increased from moderate in T1 and T2 to good in T3.

**Table 2:** AUROC results for the five optimized machine learning models.

Times	N° features	DT	XGBoost	KNN	LR	SVC
T1	1	0.78	0.74	0.69	0.69	0.72
	2	0.75	0.76	0.66	0.69	0.76
	3	0.76	**0.79**	0.64	0.68	0.66
	4	0.70	0.78	0.58	0.71	0.54
	5	0.71	0.73	0.55	0.68	0.59
T2	1	0.74	0.75	0.71	0.74	0.77
	2	0.84	0.78	0.71	0.71	0.68
	3	**0.87**	0.79	0.76	0.73	0.80
	4	0.76	0.82	0.68	0.75	0.77
	5	0.85	0.78	0.71	0.76	0.76
T3	1	0.80	0.85	0.76	0.83	0.87
	2	0.83	0.86	0.86	0.90	0.90
	3	0.87	0.87	0.86	0.89	**0.91**
	4	0.87	0.90	0.87	0.89	0.85
	5	0.86	0.90	0.85	0.89	0.91

Increasing the number of features considered in each ML model brings a modest performance gain for 3 features at T1, AUROC = 0.78 (0.68–0.90). However, the gain increases for T2, AUROC = 0.87 (0.76–0.98) and T3, AUROC = 0.91 (0.81–1.00). Once again, the best model for the multi-features scenario changed from the moderate performance of XGBoost at T1, to the good performance of DT at T2, and to the excellent performance of SVC at T3.

An important aspect to be considered is the impact of the number of features. At all time points, we observed that the increase in the number of features tends to improve the performance of the models, especially for XGBoost, which benefited most from this expansion. However, this increase in feature complexity may also have led to an increase in variance, as evidenced by the greater variation in results. On the other hand, the use of the 3 most important features stood out in all time points, suggesting that a careful selection of features may be more beneficial in some cases than including all available features.

**Figure 5:**
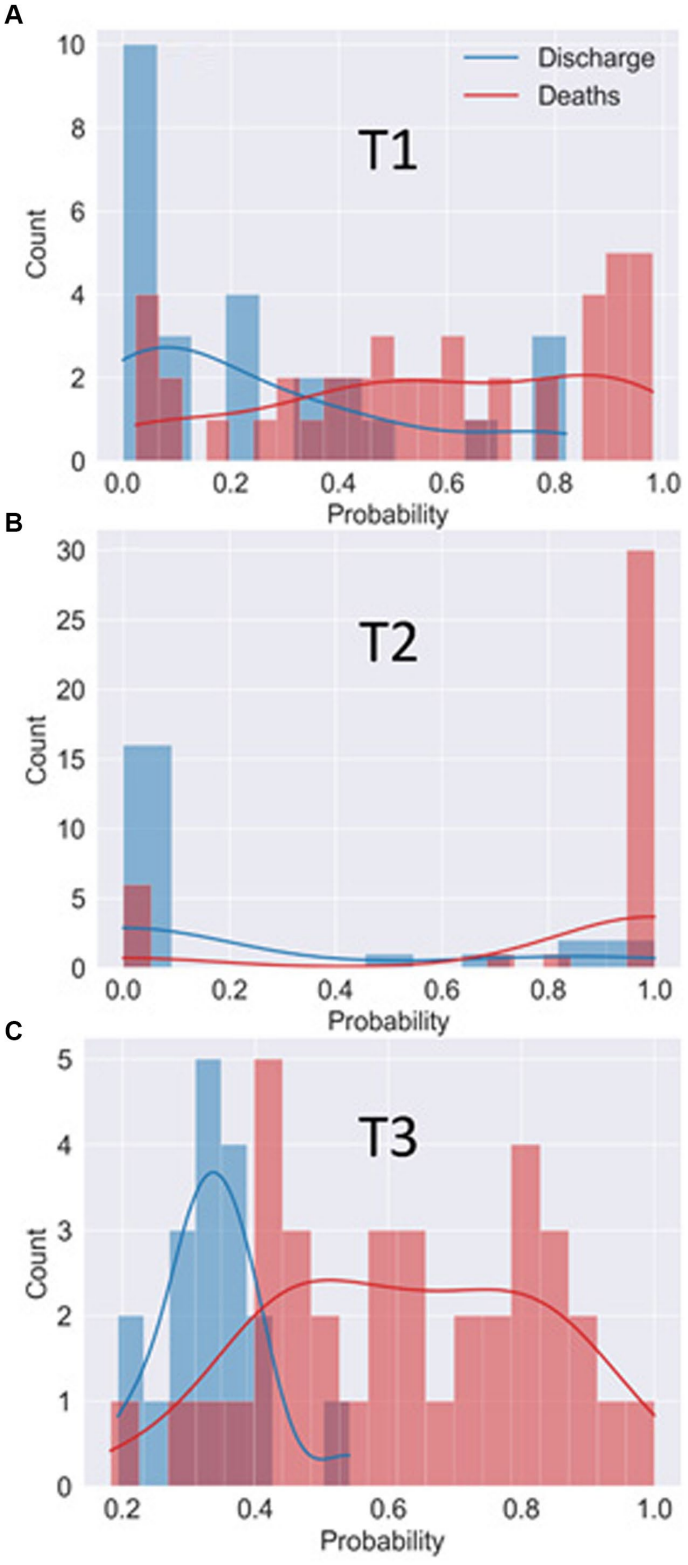
Histogram of the probability of patients being classified in the “death” class by the best model in T1 **(A)**, T2 **(B)** and T3 **(C)**. The histogram bars from the true discharge class are represented in blue while the histogram bars for the true death class are represented in red. The solid lines represent the estimated probability distribution for each class.

From the ROC curve of each of the best models at each moment, we determine the optimal threshold values that separates both classes. The optimal threshold is obtained from the point on the ROC curve closest to the coordinate [0,1] (23). Figure 5 displays a histogram of the probability of death for each patient. The histogram bars from the true discharge class are represented in blue, while the histogram bars for the true death class are represented in red. The solid lines are obtained from the Kernel Density Estimation (KDE) technique (24).

Based on the probability (of being in the death outcome) returned by the best ML model and the optimal thresholds calculated at each time point, it is possible to classify all patients into both classes and calculate different performance metrics such as accuracy, specificity and sensitivity. The resulting values of these performance metrics are summarized in Table 3. In addition to these metrics, Table 3 shows the mean AUROC of the training data set, obtained as the average of the AUROC of the trained data set in each LOOCV cycle. The mean AUROC of the training data set spans over all samples in the database and, in that sense, can be directly compared with the AUROC of the test data set.

**Table 3:** AUROC training, AUROC training, accuracy, specificity, and sensitivity of the best models at each time point.

Time point - model	AUROC training	AUROC test	Accuracy (%)	Specificity (%)	Sensitivity (%)
T1 - XGBoost	0.98	0.79	73	85	66
T2 - DT	0.99	0.87	85	95	79
T3 - SVC	0.92	0.91	89	94	86

At T1, the XGBoost model demonstrated an accuracy of 73%, with a specificity of 85% and a sensitivity of 66%. It is observed that, although the specificity is relatively high, indicating the model’s ability to identify true negatives, the sensitivity is relatively low, suggesting a limitation in the ability to identify true positives. This could be an indication that the model is inclined to classify more samples as negative, sacrificing the ability to detect positive cases.

At T2, the DT model exhibited a notable increase in accuracy, reaching 85%. Specificity also increased considerably to 95%, indicating an improvement in identifying true negatives. Additionally, sensitivity rose to 79%, demonstrating an improved ability to identify true positives. This suggests that the DT model achieved a better balance between the classification accuracy of the two classes, making it more robust at T2.

At T3, SVC achieved a remarkable accuracy of 89%. Specificity remained high, at 94%, while sensitivity increased further, reaching 86%. These results indicate that the SVC model can maintain a good ability to identify both true negative and true positive results, which is crucial for applications where the balance between these metrics is fundamental.

By analyzing AUROC results for training and testing at each time, it is possible to observe a positive and promising progression in the ability of machine learning models to effectively generalize their learnings to unseen data. This analysis reflects the continued evolution and advancement in the effectiveness of machine learning models, especially in medical diagnostic applications in which accuracy and the ability to distinguish between classes are critical.

**Figure 6:**
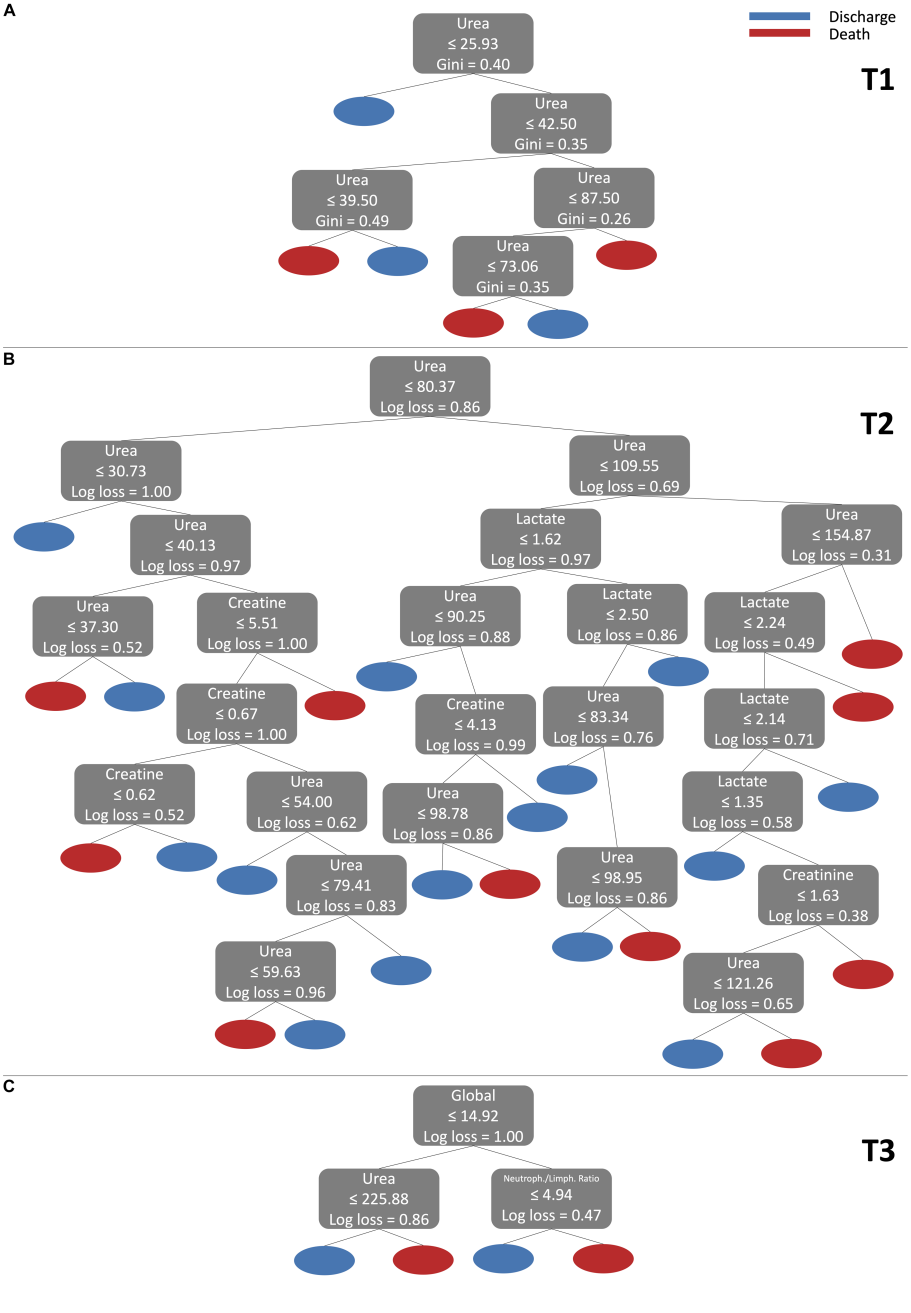
Visualization of DT model for 3 different scenarios. **(A)** refers to T1 using only one feature (Urea). **(B)** refers to T2 using 3 features (Urea, Creatine, and Lactate). **(C)** refers to T3 using 3 (Global, Urea, and neutrophil/lymphocyte sodium ratio).

The classification process carried out by ML models is usually difficult to interpret due to the mathematical complexity of the models. However, interpretable models are desirable to help physicians in the diagnosis process. To present a more intuitive and comprehensive view of the classification process, Figure 6 illustrates the DT model for 3 different scenarios. The DT model exhibits good performance in all time points, as well as a relatively straightforward interpretation. The first scenario (a) refers to time point T1 using only one feature (Urea). The second scenario (b) refers to the T2 time point using 3 features (Urea, Creatine, and Lactate). The third scenario (c) refers to the T3 time point, and 3 features were also used (Global, Urea, and neutrophil/lymphocyte sodium ratio).

For scenario (a), the decision tree begins its analysis, checking whether the value of Urea is less than or equal to 25.93. If this condition is true, the example is classified as Discharge. Then, if Urea is greater than 25.93, the tree continues the analysis. Within the range of 25.93 to 42.50 for Urea, the tree checks the value of Urea again. If Urea is within the range of 25.93 to 39.50, the example is classified as Death. On the other hand, if Urea is between 39.50 and 42.50, the classification will be Discharge. For Urea values greater than 42.50, the decision tree continues its analysis. It checks whether Urea is within the range of 42.50 to 87.50 and makes additional decisions. Within this range, if Urea is less than or equal to 73.06, the example is classified as Death. On the other hand, if Urea is between 73.06 and 87.50, the rating will be Discharge. Finally, for Urea values greater than 87.50, the decision tree classifies the example as Death. Note that the DT model finds intervals of Urea values 39,50–49,50 with a Discharge outcome. This island of Discharge outcome in the middle of a Death outcome region could be interpreted as a necessity for the model to consider more features, i.e., more processes represented by other features are influencing the outcome path of the patients at this time point. A similar description can be made for the other scenarios (b) and (c) following a similar reasoning to that made for scenario (a). Therefore, decision trees offer a clear and direct method to classify data based on feature values, thus contributing to the decision-making in a specific context, where classes (in our case Discharge and Death) represent different clinical results.

## 4 Discussion

Laboratory parameters are essential for monitoring diseases. COVID-19 is a disease that also alters several laboratory parameters and early investigation of these alterations can allow for the correct and effective treatment of the patient and maybe even prevent post-acute sequelae from the disease. Previous studies demonstrate the importance of laboratory parameters in the diagnosis of COVID-19 and that these parameters can be used to stratify patients in order to plan the appropriate treatment (25). Dwivedi et al. (26) showed in their study that crucial biomarkers such as urea, creatinine, uric acid, ferritin, C-reactive protein, LDL, fibrinogen, bilirubin, albumin and procalcitonin, as well as IL-6 were able to indicate the severity of patients with severe COVID-19. Also in this study, the authors were able to compare the biomarkers in 2 waves of COVID-19, so the parameters analyzed were higher in the second wave, while our study shows how the length of hospitalization can quickly change the hematological and biochemical parameters, which confirms the importance of using laboratory parameters to anticipate a probable outcome. The work of Chávez-Ocaña et al. (27), in addition to analyzing laboratory parameters, also analyzed interleukins. This study shows albumin, lymphocytes, platelets and ferritin as factors that may correlate with the severity of COVID-19, and with regard to pro-inflammatory cytokines, the authors found IL-6, IL-10, IL-2 and IL-17 to be elevated in severe patients. The evaluation of interleukins is interesting, however, it is a costly test, so we focused on evaluating parameters that are common in the emergency hospital routine, in addition to being more accessible and with agile results.

Many studies have addressed the importance of using artificial intelligence to diagnose or monitor patients infected with SARS-CoV-2, (28–33) using laboratory data to predict the mortality risk of patients with COVID-19. Likewise, recent reports revealed that laboratory parameters such as neutrophils, urea and respiratory indices have great unique importance in predicting patient mortality. De Souza et al. (32) shows that machine learning methods using demographic and clinical data along with comorbidities of the patients can assist in the prognostic prediction and physician decision-making. The outcome prediction in that work focuses more on individual variables such as age, symptoms, and comorbidities. Chung et al. (33) focusses the outcome prediction on the analysis of different scores. Each score involves a set of variables, with the best performance related to age, coronary heart disease, and the level of lymphocyte, procalcitonin and D-dimer. Aljame et al. (34), implemented a complex machine learning ensemble method for COVID-19 diagnosis that shows the importance of monocytes in determining positive cases of COVID-19, in addition to patients having other parameters that can diagnose the disease. Bahceci et al. (35) shows that hematological and biochemical parameters can be used to determine the patient’s treatment, as they are of low cost and accessible. Routine laboratory tests available in hospitals can be an important ally in stratifying patient severity using Machine Learning (ML) tools. ML techniques can help doctors diagnose COVID-19, complementing the results of tests such as RT-PCR and increasing the possibility of a favorable clinical outcome for the patient. The use of AI in the field of medical diagnostics fills the gap in hospitals that have limited diagnostic methods, and also speeds up medical decision-making. In addition, the use of ML allows for the analysis of various parameters, including the diversity of data, which is important in terms of the representativeness of the population studied (36–38).

Our study shows that some laboratory parameters present early changes, such as urea, for example, demonstrating that a routine hospital laboratory test can help characterize the patient who may have an unfavorable clinical evolution. Using AI tools to identify, diagnose, analyze medical images, and collect hundreds of data points quickly in hospitals could have a positive impact on the medical field. AI is also important when the diagnostic possibilities depend on many other diagnostic tools, such as sepsis, for example, which needs to combine clinical and laboratory criteria. Nevertheless, the use of IA requires care, especially in the interpretation of the results, requiring a multidisciplinary team to obtain a reliable result (38, 39). Therefore, our study highlights the importance of using tests that are already part of the laboratory routine combined with machine learning.

Predicting the clinical progression of patients with severe COVID-19 is very important because patients can present post-acute sequelae such as kidney and heart infections, liver failure and compromised lung function (40). Long COVID is tightly associated with the severe cases of COVID-19 as well as the clinical management of patients during the acute phase of disease. Considering this, improving the clinical management of acute phase patients in future waves of the disease may help in halting the Long COVID epidemics the world is experiencing in these remaining years of the pandemic. The results of our work show the parameters that are important to evaluate in patients admitted to hospitals with COVID-19, being urea and lymphocytes at early time points of acute phase taken as categorical parameters in the classification of patients who have died.

Urea, the parameter classified as the most important in the outcome of COVID-19 patients, is closely linked to the amount of protein the individual eats, i.e., the richer the protein diet, the greater the excretion of urea. The protein ingested in the diet is metabolized into essential and non-essential amino acids or into waste products and ions. In addition, amino acids are metabolized by the liver into urea, which is then excreted in the urine. The body’s protein stores can be converted into essential and non-essential amino acids or they can be metabolized to form waste products and ions, which will also be excreted in the urine. Urea is synthesized in the liver by protein catabolism and blood urea is filtered by the glomerulus and undergoes tubular reabsorption, so urea is directly related to nutritional status, protein metabolism and kidney condition. SARS-CoV-2 can activate the renin-angiotensin-aldosterone system causing renal vasoconstriction, decreased glomerular filtration and decreased urea excretion, increased absorption of water as well as sodium and passive reabsorption of urea (41, 42).

Since urea is the end product of protein metabolism, it can be used as a marker of kidney function. A study by Cheng et al. (43) tested blood urea levels combined with D-dimer as predictors of hospital mortality in COVID-19 patients. High urea levels are associated with a worse outcome in patients with heart failure. One of the reasons involved in this process is moderate to severe dehydration due to fever that ICU stay may cause, so the blood flow reaches the kidneys with less pressure, triggering damage to the renal structures. Patients undergoing mechanical ventilation have high internal pressures, which reduces venous return. This increase in pressure in the lungs reduces cardiac pressure, so if the heart cannot pump blood effectively to the kidneys and other organs, it compromises their functioning. This explains why patients with heart failure have high levels of urea, due to the inefficient functioning of the kidneys, the organs responsible for excreting urea. The study by Shaikh et al. (44) shows significant associations of biomarkers such as urea, ferritin, glucose and creatinine with mortality and ICU admission, just as our data show how urea can be a good biomarker of severity in COVID-19.

Our data shows that more severe patients with death outcome had higher concentrations of lactate and urea than patients who were discharged. We observed that these concentrations tended to increase even more in later stages. Henry et al. (45) showed in their study that high lactate values are related to a worse prognosis. Lactate dehydrogenase is an intracellular enzyme that catalyzes the interconversion of pyruvate and lactate. Severe infections can cause tissue damage mediated by cytokines and the release of lactate dehydrogenase. As this enzyme is present in lung tissues, patients with severe COVID-19 tend to release a greater amount of lactate. Thus, lactate is a predictor of worse outcomes in hospitalized patients and reflects the putative multiple organ damage and failure, that play an important role in COVID-19 patients who progress to death.

Glucose is another decisive parameter in the clinical outcome of patients with COVID-19. It is known that patients with type 2 diabetes have an increased risk of developing severe COVID-19 and according to a previous study (46), these patients have increased levels of angiotensin-converting enzyme-2 (ACE2) the receptor for SARS-CoV-2, which favors the entry of the virus and decreases its clearance. Thus, an increase in glucose is related to an increase in viral replication, a probable serious complication due to deregulation of the immune system and an increase in the inflammatory response.

The innate immune system is of great importance in viral infections, especially in respiratory infections, in which the lung is the target organ. A differential and divergent cytokine storm both systemic and in the airways will also be crucial to define immune responses and outcome of critically ill COVID-19 patients (47). This inflamed milieu also allows for improved binding to surface antigens and can influence the secretion of other cytokines as interferons and interleukins, as well as regulatory factors. In COVID-19, lymphocytes are decreased, which may suggest an inefficient IgG response and a hampered leukocyte activation (48). In this study, we found that patients who died had higher overall leukocyte counts as well as higher neutrophil percentages than patients who were discharged in the onset of acute phase. Conversely, lymphopenia was observed in COVID patients regardless of outcome. As expected, neutrophils have been abundantly studied in COVID-19 and are, therefore, expected to be a hallmark of severity. However, AI models reveal that the order of importance of these parameters diverge amongst time points, which was unexpected. At T1, only lymphocyte counts ranked second and basophils ranked in the fourth position of importance, demonstrating that leukocytes other than neutrophils need further scrutiny and may contribute for the establishment of biomarkers at early time points of disease progression. In this regard, basophils are also cells of the innate immune system that migrate to inflammatory sites during allergic inflammation and infection that triggers the production of IL-4, which stimulates the proliferation of B and T cells. The promptness of these cells to respond to an allergen may explain their order of importance in the refined AI models used here. On the other hand, at T3, neutrophil/lymphocyte ratio contributes as the second most important biomarker for assessing COVID-19 outcome, demonstrating the importance of neutrophils at late stages of disease. The study by Kaur et al. (49) reinforces our findings, by showing that lymphopenia is common in patients with COVID-19 and that severe cases of the disease at late stages in the ICU had neutrophilia. In addition, Kılıc (50) et al. shows that patients with a lower lymphocyte count associated with depletion of CD4 and CD8 T cells had an increased risk of developing a severe COVID-19 outcome. The potential mechanism for explaining this phenomena is virus-induced lysis of the lymphocytes, since these cells express ACE2 and are therefore permissive to SARS-CoV-2 (50). Cytokine-induced atrophy of lymphatic organs can also occur, which impacts on lymphocyte renewal, and another mechanism would be inflammatory pro-mediators that can induce direct lymphocyte apoptosis (49).

COVID-19 is a disease that can affect several organs and the way the host’s body reacts to the disease is fundamental in determining the patient’s outcome. Some factors are considered risk factors, such as age. Studies such as that by Chen et al. (51) show that age is the most significant risk factor for developing severe COVID-19. The results we found using ML reinforce the importance of age both in the development of the disease and in the clinical outcome of this patient. The study by Hu et al. (52) reinforces that older adult patients with comorbidities progressed to more serious illnesses, thus highlighting that the older adult were prone to developing severe acute respiratory syndrome and septic shock. Therefore, the correct diagnosis and treatment in older adult patients is crucial in order to improve survival rate and prevention of post-acute sequelae in those populational stratum.

Our study shows that the use of measurable biochemical and hematological variables (urea, lymphocytes, glucose, neutrophil/lymphocyte ratio and basophils) constitutes excellent biomarkers for the severity of COVID-19 patients and outcome prediction of hospitalized patients, with strong highlight to urea. This study shows that urea is the parameter that best classifies patient severity and rises over time, making it an important analyte to be used in machine learning algorithms to predict patient outcomes. However, in contrast to the previous studies that show the importance of age during severe COVID (51), we observed that once a patient is under treatment at the ICU, other parameters such as urea, lymphocytes, glucose and basophils at T1 were more important than age. As the patients’ hospitalization time progressed (T2 and T3), age did not appear as an important feature, as other laboratory parameters such as urea, creatinine, lactate, eosinophils, neutrophil/lymphocyte ratio and global leukocytes. Therefore, our study demonstrates the importance of machine learning algorithms in the clinical evolution of patients.

The use of ML in the clinical monitoring of patients can generate fast and efficient results, ML can also be used to predict new outbreaks, using epidemiological data (53, 54). Routine tests in the hospital environment are essential for predicting a patient’s clinical outcome, and when coupled with artificial intelligence, predictions can contribute even further to the survival rates and clinical management of patients. This work shows that laboratory parameters can change early and late during COVID-19 at its severe form, and conventional statistical analyses are insufficient to promote predictive power and contribute to decision making and clinical management of patients. Therefore, we present ML algorithms as a tool for predicting the clinical outcome of COVID-19 patients, to improve our preparedness for the more assertive and early treatment in future pandemics of newly mutated immune-resistant SARS-CoV-2 variants.

### 4.1 Limitations

Our study evaluates laboratory parameters at different times in a longitudinal design performed with patients from admission until the outcome (discharge or death), which limits the sample size of the study. The machine learning method here developed focus not only in performance, but also interpretability and generalizability of the models. However, the relatively low number of patients remains an important limitation, as well as the difficulties in obtaining a full set of data for all patients at all time points. Due to the rapid evolution of this disease, a more frequent collection of laboratory analysis (more time points) is also desirable and should be considered for future investigations.

## Data availability statement

The data analyzed in this study is subject to the following licenses/restrictions: The data contains the identification of the patients in the study. Requests to access these datasets should be directed to alicelourenco90@gmail.com.

## Ethics statement

The studies involving humans were approved by Research Ethics Committee of the Universidade Federal de Minas Gerais - Ethics Committee (CAAE: 45086721.1.0000.5149 - opinion number 4.751.423). The studies were conducted in accordance with the local legislation and institutional requirements. The participants provided their written informed consent to participate in this study.

## Author contributions

AL: Writing – original draft, Writing – review & editing. PA: Writing – original draft, Writing – review & editing. AP: Writing – original draft. GM-F: Writing – original draft. LG-dP: Writing – review & editing. CM: Writing – original draft, Data curation. FF: Writing – review & editing. JP: Writing – original draft, Writing – review & editing. JC-dR: Writing – original draft, Writing – review & editing.
