# Corrigendum: Algorithms for predicting COVID outcome using ready-to-use laboratorial and clinical data

**DOI:** 10.3389/fpubh.2024.1508116

**Published:** 2024-11-19

**Authors:** Alice Aparecida Lourenço, Paulo Henrique Ribeiro Amaral, Adriana Alves Oliveira Paim, Geovane Marques-Ferreira, Leticia Gomes-de-Pontes, Camila Pacheco Silveira Martins da Mata, Flávio Guimarães da Fonseca, Juan Carlos González Pérez, Jordana Grazziela Alves Coelho-dos-Reis

**Affiliations:** ^1^Laboratório de Virologia Básica e Aplicada, Instituto de Ciências Biológicas, Departamento de Microbiologia, Universidade Federal de Minas Gerais, Belo Horizonte, Brazil; ^2^Departamento de Física, Instituto de Ciências Exatas, Universidade Federal de Minas Gerais, Belo Horizonte, Brazil; ^3^Hospital Risoleta Tolentino Neves, Universidade Federal de Minas Gerais, Belo Horizonte, Brazil; ^4^CT Vacinas, Universidade Federal de Minas Gerais, Belo Horizonte, Brazil

**Keywords:** SARS-CoV-2, COVID-19, hematological and biochemical parameters, predictive biomarkers, machine learning

In the published article, an author name was incorrectly written as Geovane Ferreira Marques. The correct spelling is Geovane Marques-Ferreira.

The authors apologize for this error and state that this does not change the scientific conclusions of the article in any way. The original article has been updated.

